# Quantifying the binding landscapes of protein–protein interactions

**DOI:** 10.1038/s42004-021-00608-w

**Published:** 2021-11-26

**Authors:** Andrew J. Bissette

**Affiliations:** Communications Chemistry, https://nature.com/commschem

## Abstract

Small changes in protein structure can have pronounced effects on protein–protein interactions, but quantifying this has only recently become possible. Now, the binding landscapes of three homologous enzyme–inhibitor complexes are quantified and shown to depend on whether the inhibitor binds its natural target or a structurally similar protein.

Protein–protein interactions (PPIs) are important targets in medicinal chemistry, including interactions between antibodies and antigens, or enzymes and peptide-based inhibitors. But, characterising the relationship between a protein’s primary sequence and interaction strength remains a formidable challenge. Now, a collaboration led by Julia Shifman from the Hebrew University of Jerusalem, and Niv Papo from Ben-Gurion University of the Negev, reports comprehensive binding landscapes for three homologous protease–inhibitor PPIs (10.1021/jacs.1c08707)^1^.

A binding landscape describes the effect of each possible single and double mutation for a given protein on its binding affinity to another molecule, such as a receptor or inhibitor. But to obtain such a landscape requires preparing tens of thousands of mutants and quantifying their binding affinities. Shifman and colleagues previously reported a method to achieve this and used it to characterise the PPI between bovine trypsin and its inhibitor, BPTI, as a proof of principle^2^. However, while this study showed that the method can map the binding landscape of a single PPI, its potential to offer new insight into the factors governing the shape of binding landscapes remained untapped.

Now, the method has been applied to three homologous PPIs between BPTI and the serine proteases bovine trypsin, chymotrypsin, and mesotrypsin. While the structures of these complexes are similar, their binding energies differ by nine orders of magnitude, providing an opportunity to understand the differences between structurally similar complexes that vary in degree of optimisation. Analysing three PPIs in parallel generated a vast amount of data, which presented new challenges. “We had to come up with a clever way of analyzing the data and of converting sequencing data into quantitative measurements of binding affinity differences,” says Shifman. “Some mutants would appear very rarely in sequencing results and some mutants would have only partial data available.” But once processed into binding landscapes, distinct patterns of behaviour began to emerge which would previously have been difficult to observe.

Notably, the high-affinity cognate complex—that is, the complex of a protein and its natural inhibitor—between bovine trypsin and BPTI exhibited a rather different binding landscape to those of the homologous, non-cognate complexes with chymotrypsin and mesotrypsin (Fig. 1). The cognate complex is optimised, and so small mutations cause reductions in binding affinity as high as 12 kcal/mol. This effect is much less pronounced for the lower-affinity, non-cognate complexes, which BPTI has not evolved to bind. “Furthermore, the three PPIs demonstrate distinct patterns of coupling energies between two simultaneous mutations that depend not only on positions involved but also on the nature of the mutation”, explains Shifman. This coupling between mutations, called epistasis, can lead to additional enhancements (positive epistasis) or reductions (negative epistasis) in binding affinity. Mapping these couplings in the high-throughput manner was not previously possible.

**Fig. 1:**
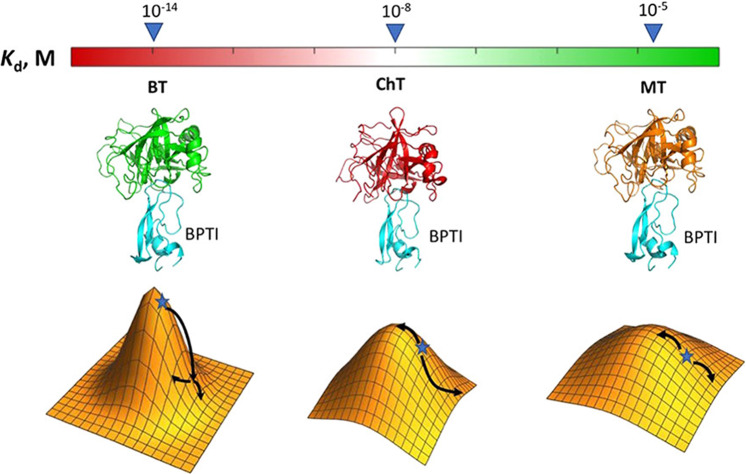
Quantifying homologous binding landscapes. The effect of single and double mutations on the binding affinity of the cognate complex between bovine trypsin (BT) and BPTI is much more pronounced than for the homologous, non-cognate complexes between chymotrypsin (CT)-BPTI and mesotrypsin (MT)-BPTI. Reprinted with permission from *J. Am. Chem. Soc*. **142**, 17261–17275 (2021). Copyright 2021 American Chemical Society.

The study provides evidence that this method for generating binding landscapes is robust and can offer insight into the structural factors governing PPIs, and is not limited to enzyme–inhibitor complexes. Future work may illuminate how the binding landscapes of other classes of PPIs, such as antibody–antigen or receptor–ligand complexes, differ from those analysed here, and may aid the development of PPI inhibitors.
